# A Neglected Gene: The Role of the *ANG* Gene in the Pathogenesis of Amyotrophic Lateral Sclerosis

**DOI:** 10.14336/AD.2024.0107

**Published:** 2024-01-07

**Authors:** Yu Zhang, Yanan Li, Shen Bin, Xi Cheng, Qi Niu

**Affiliations:** ^1^Department of Geriatrics, The First Affiliated Hospital of Nanjing Medical University, Nanjing Medical University, Nanjing, Jiangsu, China.; ^2^State Key Laboratory of Reproductive Medicine, Nanjing Medical University, Nanjing, China.

**Keywords:** Amyotrophic lateral sclerosis, *ANG* gene, Angiogenin, Pathogenesis, Genetics

## Abstract

Amyotrophic lateral sclerosis (ALS) is a rapidly progressive neurodegenerative disease with a poor prognosis. To date, more than 40 ALS-related genes have been identified. However, there is still a lack of targeted therapeutic drugs for the treatment of ALS, especially for patients with acute onset and severe disease. A series of studies reported missense heterozygous mutations with loss of function in the coding region of the *ANG* gene in ALS patients. *ANG* deficiency is related to the pathogenesis of ALS, but the underlying mechanism has not been determined. This article aimed to synthesize and consolidate the knowledge of the pathological mechanism of ALS induced by *ANG* mutation and provide a theoretical basis for ALS diagnosis and targeted therapy. This article further delves into the mechanisms underlying the current understanding of the structure and function of the *ANG* gene, the association between *ANG* and ALS, and its pathogenesis. Mutations in *ANG* may lead to the development of ALS through the loss of neuroprotective function, induction of oxidative stress, or inhibition of rRNA synthesis. *ANG* mutations and genetic and environmental factors may cause disease heterogeneity and more severe disease than in ALS patients with the wild-type gene. Exploring this mechanism is expected to provide a new approach for ALS treatment through increasing *ANG* expression or angiogenin activity. However, the related study is still in its infancy; therefore, this article also highlights the need for further exploration of the application of *ANG* gene mutations in clinical trials and animal experiments is needed to achieve improved early diagnosis and treatment of ALS.

## 1. Introduction

Amyotrophic lateral sclerosis (ALS) is a rapidly progressive neurodegenerative disease. The aetiology of ALS is still unclear, but it mainly affects the motor neurons of the brain and spinal cord, ultimately affecting respiratory function and possibly leading to death from respiratory failure within approximately three to five years after diagnosis. ALS patients may also develop overlapping symptoms, such as cognitive and behavioural changes. The pathogenesis of ALS is still being explored. The current widely recognized theory is that ALS is caused by the interaction of various pathogenic mechanisms, including protein aggregation, oxidative stress, mitochondrial dysfunction, excitotoxicity, RNA metabolism, synaptic function changes, axonal transport disorders, and neuroinflammation [1, 2]. The human nervous system comprises neurons, and those responsible for motor function are called motor neurons. Diseases in which motor neurons in the human body are damaged and gradually worsen are collectively called motor neuron diseases (MNDs), of which ALS is the most common. MNDs are a significant public health challenge. According to research statistics, approximately 330,918 people worldwide suffered from MNDs in 2016, resulting in an estimated 926,090 disabilities and 34,325 deaths [3]. The prevalence and incidence of ALS vary among different populations, especially regarding age and sex. In addition, some studies have revealed regional and temporal differences. Several researchers have summarized the global prevalence and incidence of ALS through meta-analysis [4]. The authors found that the ALS prevalence ranged from 1.57/1,00,000 to 9.62/1,00,000 person-years, and the ALS incidence ranged from 0.42/1,00,000 to 2.76/1,00,000 person-years. The prevalence and incidence of ALS are greater in developed areas. Approximately 10% of ALS patients have a history of familial ALS (FALS), which can be inherited through autosomal dominant, autosomal recessive, or X chromosome inheritance, while the remaining cases are called sporadic ALS (SALS) [5]. In recent years, with the progress of genome-wide association studies and second-generation sequencing technology, as well as the development of molecular biology, genetic research on ALS has made substantial progress.

The protein encoded by the *ANG* gene is a member of the RNase A superfamily. This protein is a potent mediator of new blood vessel formation and, in addition to the name RNase5, is commonly called angiogenin (Ang)[6]. This protein induces angiogenesis after binding to actin on the surface of endothelial cells. Ang also accumulates at the nucleolus, where it stimulates ribosomal transcription. Under stress conditions, this protein translocates to the cytosol, where it hydrolyses cellular tRNAs and influences protein synthesis. A signal peptide is cleaved from the precursor protein to produce a mature protein that contains a nuclear localization signal, a cell binding motif, and a catalytic domain. Ang has been shown to be both neurotrophic and neuroprotective, and the mature protein has antimicrobial activity against some bacteria and fungi. Due to its effect on rRNA production and angiogenesis, *ANG* plays important roles in cell growth and tumour progression[7]. In recent years, an increase in research on *ANG* has indicated that Ang may be involved in the pathogenesis of ALS. A study by Conforti *et al*. [8] reported a correlation between the promoter of the vascular endothelial growth factor gene and the predisposition to SALS in Northern European ALS patients. Greenway [9] reported that the *ANG* single-nucleotide polymorphism (SNP) rs11701 was also associated with disease susceptibility in patients with ALS. Subsequently, Greenway *et al*. also detected *ANG* mutations in FALS and SALS patients, and Ang became the second angiogenic factor associated with ALS. In 2006, *ANG* was officially recognized as an ALS disease-related gene (ALS 9) [9]. However, the pathogenesis of ALS caused by *ANG* has not been elucidated.

In this article, we elaborated on the structure and function of the *ANG* gene. We summarized the current research status on *ANG* and ALS to further explore the possible pathogenesis of *ANG* gene-related diseases. Finally, we highlighted the current clinical trials and animal studies on the *ANG* gene. However, there are still some controversies in the current research, and additional clinical and animal studies are needed to explore the pathogenesis of *ANG* in ALS to achieve improved early diagnosis and treatment.

## 2. Challenges: The genetic and clinical heterogeneity of ALS leads to a limited understanding of the pathogenic mechanisms of *ANG*

### Genetic Heterogeneity

ALS is a genetically heterogeneous disease with multiple causative genes. In recent years, with the progress of genome-wide association studies and second-generation sequencing technology, as well as the development of molecular biology, genetic research in ALS has made substantial progress. To date, nearly 40 genes have been confirmed to be related to the incidence of ALS [10], and the most commonly studied genes include superoxide dismutase 1 (*SOD1*), trans-activation reaction DNA-binding protein (TAR DNA binding protein 43, TPD-43) (*TARDBP*), sarcoma fusion protein (*FUS*), optineurin (*OPTN*), sequestosome 1 (*SQSTM1*), angiogenin (*ANG*), D-amino acid oxidase (*DAO*), dynactin subunit 1 (*DCTN1*), VAMP-associated protein B and C (*VAPB*), sigma nonopioid intracellular receptor 1 (*SIGMAR1*), granulin precursor (*GRN*), and C9orf72-SMCR8 complex subunit (*C9orf72*) [10, 11]. Due to differences in genetic background, the spectrum of pathogenic genes for ALS varies among different countries or regions. Especially between Europe and Asia, there are significant differences in the mutation frequencies of these ALS genes. In the European population, the most common mutations are *C9orf72* repeat expansions (FALS 33.7%, SALS 5.1%), followed by SOD1 (FALS 14.8%, SALS 1.2%), *TARDBP* (FALS 4.2%, SALS 0.8%), and *FUS* gene mutations (FALS 2.8%, SALS 0.3%). In contrast, in the Asian population, the most common mutations are SOD1 gene mutations (FALS 30.0%, SALS 1.5%), followed by *FUS* (FALS 6.4%, SALS 0.9%), *C9orf72* (FALS 2.3%, SALS 0.3%), and *TARDBP* (FALS 1.5%, SALS 0.2%) gene mutations [12]. Studies on ALS pathogenic genes have focused on *SOD1*, *FUS*, *TARDBP*, and *C9orf72* as the first echelon of genes of interest, while there are relatively few studies on other rarer genes [5, 10, 11]. Since 2006, *ANG* has been identified as an essential gene for ALS [9] and is referred to as the ALS9 gene. In the ALS population, 33 different missense mutations in the *ANG* coding region have been identified [13-15]. The proportion of ALS patients with *ANG* mutations ranged from 0% to 2.6%, which may be related to race, sample size, or the proportions of FALS and SALS patients. Therefore, *ANG* appears to be the second most common mutated gene in ALS (second only to *SOD1*) and seems to be the first loss-of-function gene identified in ALS to date [15]. As a result, exploring the mechanisms by which the *ANG* gene contributes to the onset and progression of ALS is crucial for its diagnosis and treatment.

### Variable Penetrance

*ANG* gene variants were first found to be associated with ALS in patients in Scotland, Ireland, the United Kingdom, and Sweden [16]. Subsequent studies have also identified *ANG* mutations in ALS patients in Italy, France, Germany, the Netherlands, Belgium, Hungary, China, and India [8, 16-18]. Several *ANG* variants that have been identified affect the ribonuclease activity of Ang due to their proximity to the catalytic sites of proteins [15]. Studies have shown that *ANG* variants can inhibit the shuttling of Ang between the nucleus and cytoplasm [15] and reduce the stability of the protein [17]. Subsequent studies have shown that Ang plays a neuroprotective role in in vitro models of motor neuron injury induced by excitotoxicity and hypoxia, including in dopaminergic SH-SY 5Y neuroblastoma cells [19, 20]. When *ANG* is overexpressed at similar levels in neurons, many *ANG* variants have reduced neuroprotective activity compared to wild-type (WT) *ANG* [19]. However, it is unclear which cells in the nervous system are susceptible to *ANG* mutations, which lack specificity. For example, *ANG* variants have also been identified in familial Parkinson’s disease (PD) patients from Germany, the Netherlands, and Italy [21, 22]. The frequency of the PD-*ANG* variant was highly similar in both previous studies (0.45%/0.47%) compared to the control group (0.04%/0%). In addition, Van Es *et al*. reported a similar frequency of *ANG* variants in ALS patients and PD patients (0.46%/0.45%, compared with 0.04% in the control group) [21]. In recent studies, several of these *ANG* variants have been shown to reduce ribonuclease activity compared to that of WT *ANG* individuals [23].

**Table 1:** Variations in the *ANG* gene in ALS patients.

Rank	Mnemonic	Chromosome	Position hg19	HGVS	Reference
**1**	A24T	14	21161793	NM_001145.4:c.70G>A	[28]
**2**	c.*2C>T	14	21162169	NM_001385274.1:c.*2C>T	[13]
**3**	c.*6G>A	14	21161718	NM_001145.4:c.*6G>A	[29]
**4**	C39W	14	21161912	NM_001145.4:c.189C>G	[30]
**5**	D46G	14	21161860	NM_001145.4:c.137A>G	[29]
**6**	F100I	14	21162021	NM_001145.4:c.298T>A	[30]
**7**	F12F	14	21161759	NM_001145.4:c.36C>T	[30]
**8**	F12S	14	21161758	NM_001145.4:c.35T>C	[30]
**9**	G15D	14	21161767	NM_001145.4:c.44G>A	[30]
**10**	G44G	14	21161855	NM_001145.4:c.132C>G	[13]
**11**	H138R	14	21162136	NM_001145.4:c.413A>G	[30]
**12**	H84R	14	21162046	NM_001145.4:c.323A>G	[31]
**13**	I70V(I46V)	14	21161931	NM_001145.4:c.208A>G	[25]
**14**	K17I	14	21161845	NM_001145.4:c.122A>T	[32]
**15**	K41I	14	21161973	NM_001145.4:c.250A>G	[14]
**16**	K64I	14	21161914	NM_001145.4:c.191A>T	[14]
**17**	K78E	14	21161955	NM_001145.4:c.232A>G	[32]
**18**	K84E	14	21161973	NM_001145.4:c.250A>G	[33]
**19**	L59P	14	21161899	NM_001145.4:c.176T>C	[33]
**20**	M1I	14	21161726	NM_001145.4:c.3G>A	[8]
**21**	N73S	14	21161941	NM_001145.4:c.218A>G	[34]
**22**	P136L	14	21162130	NM_001145.4:c.407C>T	[30]
**23**	P21S	14	21161784	NM_001145.4:c.61C>T	[30]
**24**	Q36L	14	21161830	NM_001145.4:c.107A>T	[30]
**25**	R145C	14	21162156	NM_001145.4:c.433C>T	[35]
**26**	R145H	14	21162157	NM_001145.4:c.434G>A	[30]
**27**	R31K	14	21161887	NM_001145.4:c.164G>A	[30]
**28**	S52N	14	21161878	NM_001145.4:c.155G>A	[30]
**29**	T104S	14	21162034	NM_001145.4:c.311C>G	[30]
**30**	V127I	14	21162102	NM_001145.4:c.379G>A	[31]
**31**	V137I	14	21162132	NM_001145.4:c.409G>A	[13]
**32**	W89X	14	21162061	NM_001145.4:c.338G>A	[36]
**33**	Y38H	14	21161835	NM_001145.4:c.112T>C	[29]

Currently, a total of 19 *ANG* missense mutations have been found worldwide [14], and ALS patients with *ANG* mutations account for approximately 0.86% of all ALS patients, 2.0% of FALS patients, and 0.7% of SALS patients [13, 24]. In one study, researchers identified a novel missense mutation at *ANG* coding location 191 (A to T), which resulted in Lys being replaced by Ile (K40I), in 2 of 169 Irish ALS patients; however, this mutation was not found in 171 control subjects [9]. Subsequently, through sequence screening of 1629 ALS patients, 7 heterozygous missense mutations in the *ANG* coding region were identified in 15 patients [14], with an overall frequency of approximately 1%, and the frequency in FALS patients (4/259, 1.5%) was greater than that in SALS patients (11/1370, 0.8%). Another seven mutations were found in 9 of 737 Italian ALS patients [13]. *ANG* mutations in the Italian population also appear to separate FALS (3/132, 2.3%) from SALS (6/605, 1%), with an overall frequency of 1.2% [13]. Among 3170 ALS patients from populations in Ireland, Scotland, Sweden [14], North America [14], and Italy [13], a total of 14 missense mutations were identified in the *ANG* coding region at 13 locations. Of these mutations, three occurred in the signal peptide region, and 11 occurred in the mature protein. In seven sequencing studies (including 3,003 healthy controls), two *ANG* mutations were identified in non-ALS controls [13]. The first, the K17I mutation, was found in a healthy 65-year-old man of European descent. The second, the I46V mutation, was found in 11 of 1568 healthy Italian controls [13, 25]. Thus, the I46V mutation does not appear to be associated with ALS in Italian patients but does appear to be associated with ALS in Scottish patients; 3 out of 398 ALS patients carried the mutation, but none of the 299 controls carried it [14]. The *ANG* gene mutations in ALS patients are shown in Table 1.

*ANG* appears to be the second most commonly mutated gene associated with ALS [26] after *SOD1*. The proportion of ALS patients with *ANG* mutations ranged from 0% to 2.6%, which may be related to race, sample size, or the proportions of FALS and SALS patients. The *ANG* mutations found in FALS patients were all inherited as autosomal dominant genes. In the study by Greenway [14], the average age of onset was 56 years, among which the greatest proportion of patients exhibited symptoms spreading to the medulla. However, in other studies, most ALS patients with *ANG* mutations presented with limb weakness and atrophy, and the age of onset was older than 50 years [13]. In addition, one SALS patient with a G20G synonymous mutation, I46V mutation, and K54E mutation had frontal dementia, and one FALS patient with a K17I mutation had frontotemporal dementia (FTD), suggesting that *ANG* mutations may be related to cognitive impairment [27].

### *ANG* structure and angiogenin function

Located on chromosome 14q11, the *ANG* gene is 5.4 kb in length and consists of two exons, but only exon 2 has a coding function [37]. The protein encoded by *ANG* is a member of the pancreatic ribonuclease superfamily, and the mature protein is composed of 123 amino acids with a relative molecular weight of approximately 14100 kDa [17, 38-40]. *ANG* has the following functional domains: a signal peptide, a catalytic centre with ribonuclease activity, and a nuclear localization sequence (NLS) at amino acid residues 31-35, which mainly mediates human nuclear transport. In addition, one receptor-binding domain and one binding domain are related to immune regulation [18]. Existing studies have shown that Ang is expressed in human endothelial cells, glial cells, and motor neurons of the spinal cord and dorsal root ganglia in embryonic and adult stages. Induced by hypoxia, Ang can regulate the neovasculature, promote tRNA transcription and ribosome biosynthesis, and thus promote the axonal growth of motor neurons. It also protects motor neurons from death caused by hypoxia [41].

Ribonucleases (RNases) are ubiquitous enzymes that play important roles in transcription and translation. By catalysing the cleavage of RNA, RNases can oversee numerous biological processes [42]. RNase H degrades the RNA primers that facilitate DNA replication; tRNA used for protein synthesis by the ribosome is matured by RNase P; and miRNA, which regulates the expression of other RNA transcripts, is processed by Dicer and Drosha. These functions are highly conserved and essential for eukaryotic cells [43]. Vertebrates express a unique RNase superfamily called vertebrate-specific secreted or pancreatic-type RNases (ptRNases). Ang is a vertebrate-specific ptRNase. Humans have only one *ANG* gene, while mice have 5 *ANG* orthologues and 3 pseudogenes [44]. The *ANG* gene is absent in some mammalian genomes (e.g., guinea pigs and dogs). Ang is a secreted molecule [45]. Ang (also known as RNase 5) is a protein that belongs to the vertebrate secretory ribonuclease superfamily. Other members of this family include RNase 1 (pancreatic ribonuclease), RNase 2 (eosinophil-derived neurotoxin), RNase 3 (eosinophil cationic protein), RNase 4, RNase 6 (k6), RNase 7, and RNase 8 [46]. In contrast to most members of this family, Ang exhibits relatively low ribonuclease activity. Ang was first discovered in human colorectal adenocarcinoma cells, and subsequent studies have reported its role in angiogenesis, cancer, ischaemia, and infection [46]. Ang stimulates endothelial cell proliferation and is needed for the angiogenic activity of vascular endothelial growth factor (VEGF) and fibroblast growth factor-2 (FGF-2) [47]. VEGF and FGF-2 activate protein synthesis through tyrosine kinase receptors, thereby increasing ribosomal RNA (rRNA) transcription [48]. Ang works synergistically with VEGF and FGF-2 to increase protein synthesis in endothelial cells and is needed for cell proliferation [49]. Ang-induced RNA transcription also requires Ang ribonuclease activity and occurs through epigenetic activation of *ANG* promoters [50, 51]. Studies have also shown that Ang indirectly stimulates the phosphoinositide-3-kinase (PI3K) and Akt pathways in endothelial cells and bladder cancer cells [52-54]. Under stress conditions, such as oxidative stress and disruption of protein balance, Ang can accumulate in cytosolic stress granules [55, 56]. Stress particles are cytoplasmic lesions containing untranslated mRNAs and RNA-binding proteins that are produced by cellular stress and act to prevent protein translation [57-59]. Ang can be taken up into the cytoplasm of target cells through endocytosis [60], which occurs mainly through paracrine signalling. Since Ang is secreted from cells, several studies have investigated Ang receptors and cellular uptake mechanisms. Ang is a surface receptor ligand of the plexin family. Yu *et al*. reported that Plexin-B2 can act as a receptor for Ang in endothelial cells, neuronal cells, tumour cells, normal haematopoietic and leukaemia stem cells, and progenitor cells [61]. Plexin-B2 is expressed in the postnatal and adult nervous systems, especially in subventricular zone (SVZ)-derived neural stem cells [62]. However, other mechanisms can also absorb Ang into other cell types. Studies have shown that exogenous or neuronal Ang can be effectively taken up by astrocytes [58] and alter their secretomes [63]. The role of astrocytes in neurodegenerative diseases, including PD and ALS, is increasingly being elucidated [64, 65].

### Limited understanding of the pathogenesis of *ANG*-induced ALS

#### Polygenic inheritance

With the discovery of an increasing number of ALS-related genes, in-depth study of the pathogenesis of ALS has suggested that it may be a polygenic inherited disease. In 2012, van Blitterswijk [66] reported an oligogenic inheritance pattern in FALS patients. In one FALS family, most patients carried both *ANG* and *TARDBP* mutations. Subsequent mass screenings of most ALS-related genes revealed that some SALS patients can also carry 2 or more ALS-related gene mutations. In 2016, Bury [67] also observed an ontogenetic phenomenon involving two genes, *OPTN* and *C9orf72*, in FALS patients. Oligogenic inheritance is similar to polygenic inheritance but involves fewer genes. Phenotypic effects depend on the accumulation and interaction of several genes. Clinically, the phenotypes of ALS patients are different, and there are significant differences in the age and site of onset. ALS-related gene mutations can also be detected in other neurological degenerative diseases. For example, patients with FTD may carry mutations in ALS-related genes such as *TARDBP*, *FUS*, *OPTN*, *VCP*, and *C9orf72* [68, 69], and patients with the same *TARDBP* mutation in the same family may present with ALS and PD [70]. Mutations in *ANG* have also been detected in patients with PD [30]. Abnormal repeats of GGGGCC nucleotide hexamers in the *C9orf72* gene have also been found in patients with Alzheimer's disease [71]. These phenomena may result from several different gene mutations acting together. In epidemiology, several studies have reported that ALS exhibits a similar phenomenon of family aggregation as seen in polygenic genetic diseases. These results suggest that the pathogenesis of ALS may involve multiple genes and different signalling pathways.

*ANG* mutations can coexist with mutations in other genes. Millecamps *et al*. [32] reported that 1 FALS patient had both the *ANG* K17I mutation and the *FUS* R52IC mutation, and another FALS patient had both the *ANG* K54E mutation and the *FUS* R52IS mutation. However, gene mutation and disease phenotype codissociation were observed for the K54E and K17I mutations, suggesting that these two *ANG* mutations may not be pathogenic genes in these two ALS patients. Penco *et al*. [26] reported that, in a family with the C93D mutation of the *SOD1* gene, the age of onset and the degree of disease progression significantly differed between the proband and other mutation carriers. Further screening revealed that the proband also carried the *ANG* IVS1+27C/T mutation. The researchers speculate that the genetic background of the IVS1+27C/T mutation may be why the progenitors developed the disease earlier and more rapidly than other carriers of the *SOD1* G93D mutation. Luigetti *et al*. [35] also described a case of a SALS patient with both a *SOD1* G93D mutation and an *ANG* R121C mutation whose disease progressed more rapidly than that of other carriers of the G93D mutation, and the patient died of respiratory failure 2 and a half years after onset. Surprisingly, the pathogenic R121C mutant displays greater ribonuclease activity than that of the WT [23]. Furthermore, this mutant seems to be more inclined to dimerize than the WT and the S28N mutant, the latter of which is already known to be inclined to dimerize [15]. It was confirmed that *ANG* gene mutations could affect the clinical manifestations of *SOD1* mutant gene carriers. These results suggest that *ANG* may be a modifier of ALS, and these modifiers and environmental factors may be responsible for the heterogeneity of the clinical phenotypes of carriers of the same mutant gene.

#### Loss of ANG neuroprotective function

**Figure 1.**
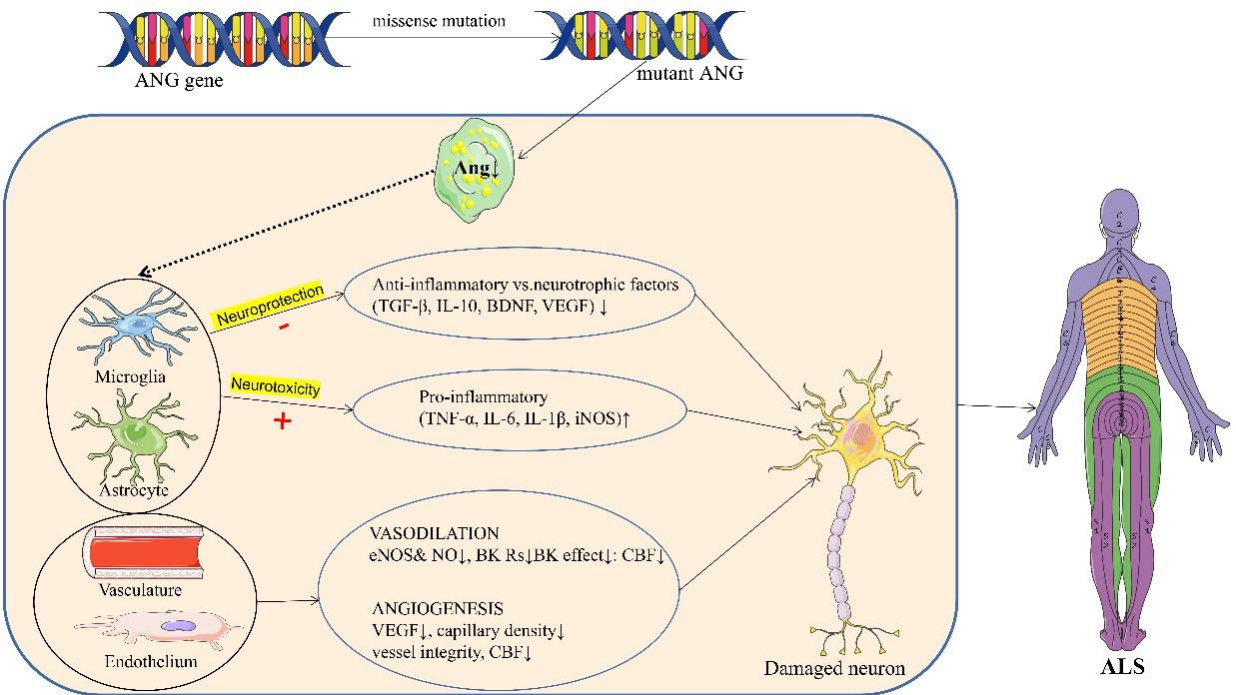
**Mutant *ANG* attenuates neuroprotective effects**. This review shows that neuroinflammation has an essential role in ALS, with microglial and astrocyte-mediated neuroinflammation as its prominent feature. These neural cells have both neuroprotective and detrimental effects. *ANG* promotes nerve cell-mediated neuroprotection. When the *ANG* gene is mutated, this neuroprotective effect is weakened, while the damaging effect is enhanced, which induces neuroinflammation and promotes the development of ALS. In addition, Ang can also be expressed in the vasculature, promoting vasoconstriction and diastolic dysfunction and further aggravating neurological damage. Parts of the figure are adapted from SMART (https://smart.servier.com) and licenced under a Creative Common Attribution 3.0 Generic licence. Ang: angiogenin.

Several SNPs of the *ANG* gene are associated with susceptibility to ALS. Greenway *et al*. [14] reported that the rs11701 SNP of *ANG* could increase susceptibility to SALS in Irish and Scottish people but had no correlation with susceptibility to ALS in American, English, or Swedish people. Since then, studies in Italy, France, and Germany have not replicated the link between rs11701 and ALS susceptibility. However, recent studies by McLaughlin *et al*. [72] revealed that rs11701, rs9322855, rs8004382, rs4470055, and rs17114699 can increase the susceptibility to ALS in Irish people, and rs17114699 can also increase the susceptibility to ALS in Swedish people. However, none of these SNPs were associated with susceptibility to ALS in Polish people, confirming that these SNPs are associated with susceptibility to ALS in Irish people. These findings suggest differences in ALS susceptibility among ethnic groups [72]. Currently, functional studies on signal peptide mutations are lacking, and it is speculated that these mutations may affect the maturation, subcellular localization, and secretion of Ang [13]. Mature Ang depends on the two main functional domains and the NLS of its catalytic sites to remain intact and perform normal physiological functions. Most *ANG* mutations can affect these two functional domains, resulting in varying degrees of reduction in normal physiological function [13]. The ribonuclease activity of Ang proteins with the Q12L, K17I, K17E, S28N, C39W, K40I, I46V and P112L mutations decreased significantly, ranging from < 1% (K40I) to 19% (K17E) of the activity of the WT protein. Analysis of the Ang crystal structure revealed that the H114R and R121H mutations can also affect the formation of catalytic centres, which may reduce the activity of ribonucleases [73]. The nuclear transport capacity of Ang proteins with the K17I, S28N, and P112L mutations was significantly reduced or even absent, and the angiogenic activity of Ang proteins with the Q12L, K17I, S28N, C39W, K40I, and P112L mutations disappeared. In addition, WT Ang protected motor neurons from hypoxia-induced neuronal death. Moreover, the Q12L, C39W, and K40I mutations resulted in a reduced ability to promote neurite growth, loss of neuronal protective function, and even motor neuron toxicity, inducing the degeneration of motor neurons. The survival of *SOD1* G93A mutant mice was prolonged by injecting human recombinant Ang. The pathogenesis of *ANG* gene mutations is mainly due to loss of function (Fig. 1) [39].

**Figure 2.**
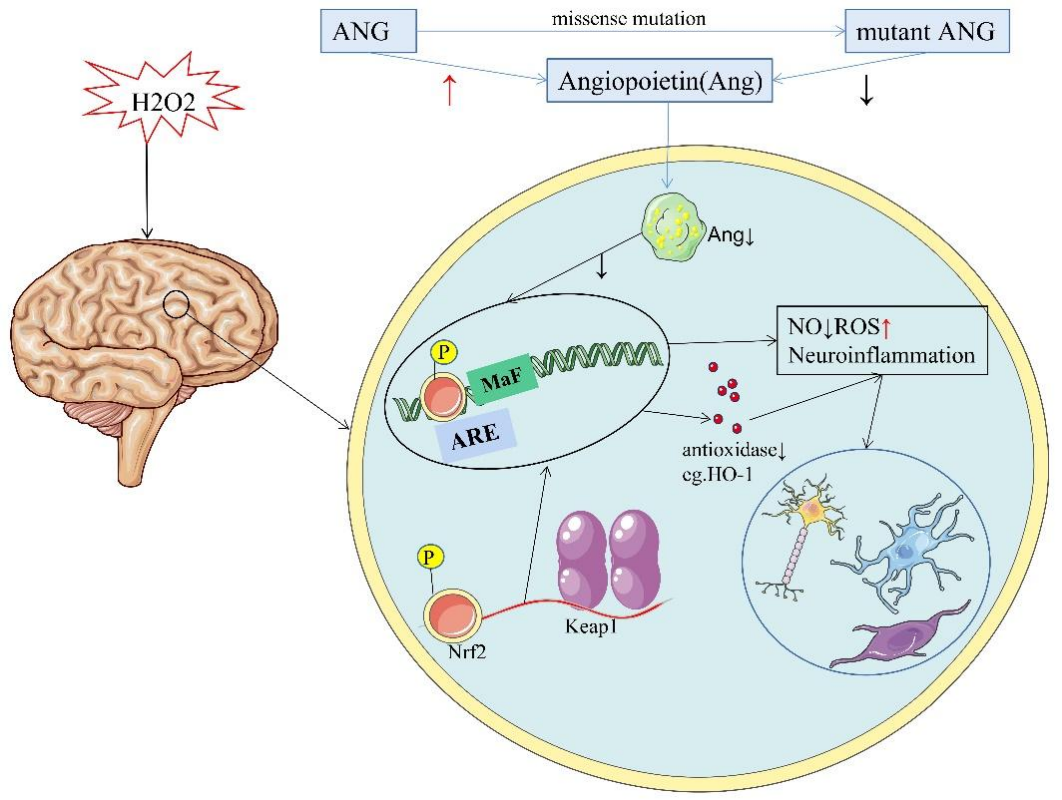
**Mutant *ANG* promotes oxidative stress**. When oxidative stress occurs, the dimeric multidomain protein Keap1 can bind to Nrf2 and increase the expression of antioxidant genes, promoting their ubiquitination and proteasomal degradation to combat cellular oxidative damage. When *ANG* is mutated, the generation of antioxidant enzymes decreases, causing cell damage and neuropathy.

Most ALS-associated *ANG* mutations are located in or near the NLS, catalytic residues, or signal peptides, resulting in the loss of ribonucleolytic activity or nuclear translocation [17]. In addition to its role in neuroangiogenesis, Ang is an influential neurotrophic factor that regulates neurogenesis, neuronal survival, and migration [74]. Ang is highly expressed in the developing nervous system in both the brain and spinal cord and promotes synaptic growth [75]. In adults with MNDs, Ang-mediated protection against stress-induced damage is dependent on Ang endocytosis in astrocytes and the subsequent adaptive modification of astrocyte transcriptomic and proteomic profiles. This neuroprotective effect was further demonstrated in the *SOD1* G93A mouse model, even at the symptomatic stage, by a prolonged mouse lifespan and improved motor performance after Ang injection [19, 76].

#### Oxidative stress

Ang belongs to the pancreatic-type RNase (ptRNA enzyme) superfamily. This secreted protein can enter cells and catalyse the cleavage of the anticodon ring of mature tRNA to produce 5' and 3' fragments, known as tRNA-derived stress-induced RNA (tiRNA) [77]. Five beta-tiRNAs recruit the translation-silencing protein YB-1 and isolate the eukaryotic translation initiation factor 4G/A complex to inhibit translation. Specific 5β-tiRNAs also trigger the assembly of stress particles at Ang localization sites [78]. Translation inhibition is critical for overcoming oxidative stress, a hallmark of neurological disorders [79]. Oxidative stress is caused by an imbalance in the production and detoxification of free radicals from reactive oxygen species (ROS) [80]. To neutralize ROS toxicity, cells are supplemented with antioxidants by activating nuclear factor erythrocyte 2-associated factor 2 (Nrf2) [81]. The dimer multidomain Kelch-like ECH-related protein 1 (Keap1) can bind to Nrf2 and promote its ubiquitination and proteasome degradation. The oxidant loses its ability to degrade Nrf2 after the sulfhydryl reaction with Keap1. Thus, Nrf2 enters the nucleus and forms a heterodimer with the small Maf protein. The heterodimer binds to antioxidant response elements to drive the expression of antioxidant enzymes, which compensate for the physiological and pathophysiological consequences of antioxidant exposure [82]. Hybridization of astrocytes from mice selectively overexpressing the *Nrf2* gene in two ALS mouse models produced double-transgenic mice, which had a more prolonged onset and survival than single-transgenic ALS mice [83]. In addition, activation of the Nrf2 pathway in astrocytes increases neuronal survival [84]. This synergy between astrocytes and neurons is similar to the Ang-mediated aspect of neuroprotection. Ang is enriched in motor neurons and protects them from ALS-related damage, such as excitotoxicity, hypoxia, and endoplasmic reticulum (ER) stress (Fig. 2) [85].

#### Inhibition of rRNA synthesis

Ang undergoes nuclear translocation, mainly in endothelial and cancer cells [86]. Nuclear translocation of Ang occurs [87] through receptor-mediated endocytosis, and this process is independent of lysosomal processing and the microtubule system [88]. Ang can bind to the promoter region of rDNA and stimulate transcription by rRNA [48, 89]. Ribosome biogenesis involves rRNA transcription, rRNA precursor processing, and mature rRNA assembly with ribosomal proteins [90, 91]. The synthesis of rRNA is the rate-limiting step in ribosome biosynthesis. Thus, rRNA transcription is essential for individual growth and development. Furthermore, rRNA transcription is also important for maintaining normal cellular function, as proteins are needed for essentially all cellular activities. Motor neurons have long axonal transport and thus robust ribosome biosynthesis.

Ang plays an essential role in maintaining the vasculature integrity of the spinal cord. Heterozygous missense mutations in the coding region or other genetic and environmental factors can cause decreased *ANG* expression, which may lead to vascular abnormalities and defects in the motor neuron environment. In addition, Ang may play a direct role in the physiological process of motor neurons, and its deficiency may accelerate or cause motor neuron degeneration. *ANG* is expressed in human and mouse spinal cord motor neurons during development and adulthood [14]. Ang deficiency can lead to inadequate ribosome biosynthesis and improper mRNA translation. The local translation of asymmetrically located mRNAs in neurons has been found to play an important role in neuronal polarity and synaptic plasticity [92]. Abnormal mRNA formation and RNA processing errors in excitatory amino acid transporter 2 were found only in neuropathologically affected ALS patients but not in other brain regions [93]. Motor neurons are the body's longest cells, so RNA processing may play a crucial role in their development, nerve repair, and regeneration. Ang is a member of the pancreatic RNase family, and RNase activity is needed for its biological activity [94]. Thus, a link between RNA processing and neurodegeneration has been established [95].

**Figure 3.**
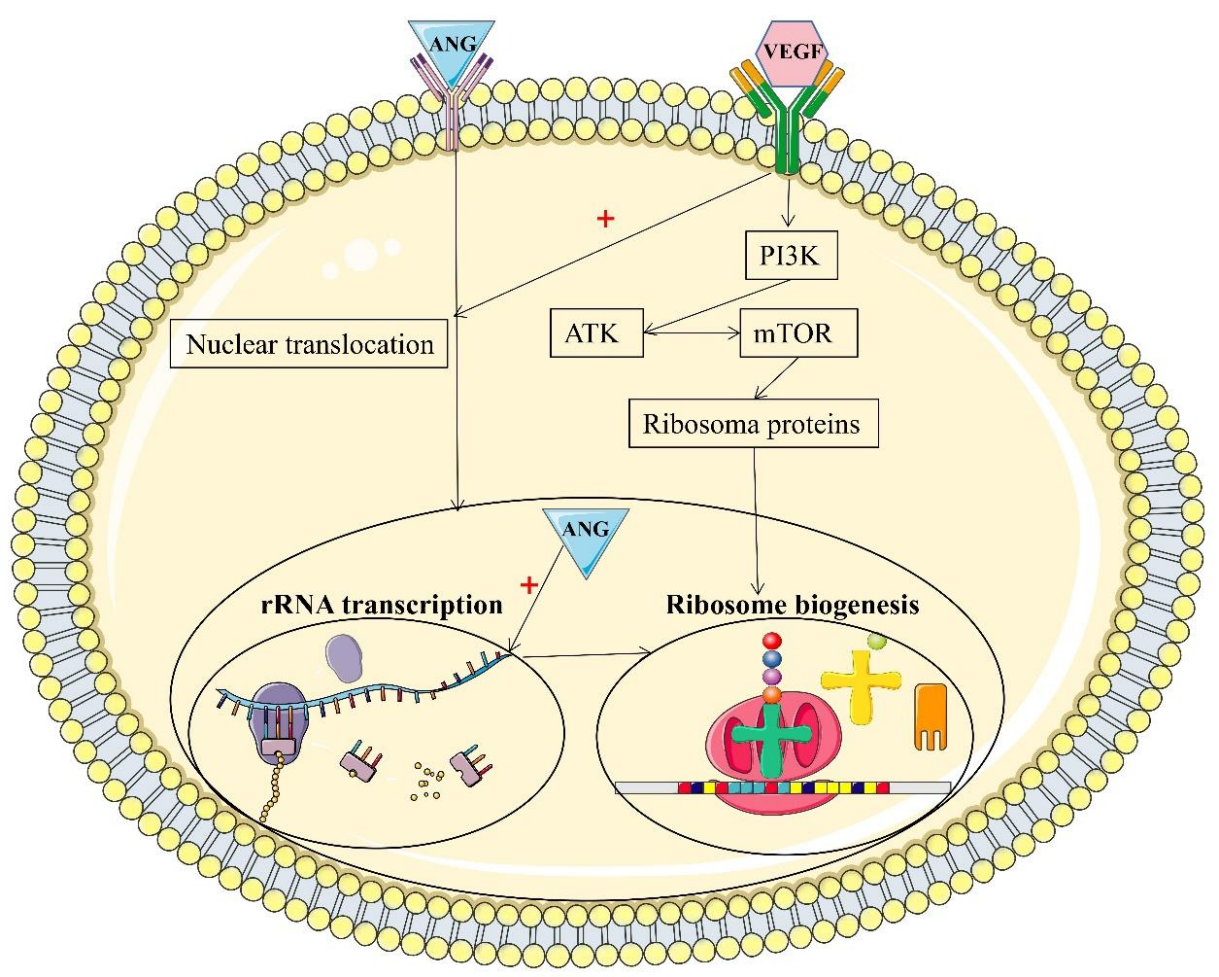
***ANG*-stimulated rRNA transcription**. Ribosome biogenesis involves rRNA transcription, precursor rRNA processing, and mature rRNA assembly with ribosomal proteins. Angiogenin translocates to the nucleus, enhancing rRNA transcription and enabling ribosome biogenesis.

All the mutated Ang proteins associated with ALS, except those with the R31K mutation, exhibit severely impaired ribonucleolytic activity. The mutant Ang proteins exhibit <1% (K40I) to 19% (K17E) of the activity of the WT protein. R31K Ang has 69% of the enzyme activity of WT Ang [25]. The *ANG* mutation identified in ALS patients is associated with a functional loss of angiogenic activity of the Ang protein. WT Ang can stimulate neurite growth and motor neurons derived from P19 embryonic cancer cells [75]. WT Ang also protects P19-derived motor neurons from hypoxia-induced cell death, but ALS-associated mutant Ang proteins (Q12L, C39W, K40I) lack this neuroprotective effect [20]. In addition, these mutant Ang proteins are cytotoxic to P19-derived motor neurons and induce their degeneration, suggesting that *ANG* mutations may be pathogenic factors in ALS (Fig. 3) [20].

### Disease Modelling

Developing accurate disease models that recapitulate *ANG*-related ALS pathogenesis in animals or in vitro is challenging. These models are essential for studying disease mechanisms and testing potential therapies.

Some of the possible mechanisms of *ANG*-induced ALS have already been discussed, and the expression of *ANG* in the spinal cord of foetuses (ranging from 15 to 30 weeks of gestation) and adults can be detected using immunohistochemistry. Ang expression in the spinal cord appears to be downregulated as development progresses but remains strongly expressed in adulthood. Intense Ang staining was observed in ventral horn motor neurons in foetal and adult patients [15]. Cytoplasmic solid and nuclear accumulation of Ang in prenatal and adult spinal motor neurons suggest a physiological role of Ang in early development and late adulthood. These findings support the hypothesis that *ANG* mutations are associated with ALS pathology. Dual immunofluorescence using the anti-Ang monoclonal antibody (mAb) 26-2F and anti-Willebrand factor polyclonal antibodies revealed that Ang was also localized to spinal endothelial cells, suggesting that Ang plays a role in maintaining the integrity of the spinal vasculature and is essential for the physiological health of motor neurons. Therefore, Ang abnormalities may play a dual role in ALS by directly affecting motor neuron function and indirectly affecting endothelial cells and abnormal angiogenesis in the spinal cord. In addition, Ang is strongly expressed in mouse brains and spinal cords [96, 97]. *ANG* expression is vital in the mouse brain and spinal cord 9.5 days after mating (pc) [75]. On Day 11.5 pc, *ANG* remained highly expressed in the telencephalon, midbrain, spinal cord, spinal ganglia, and choroid plexus. Until the second trimester, *ANG* is expressed more strongly in the nervous system than in any other tissue.

While ALS has been modelled in various organisms, additional research is needed in animal models with *ANG* gene mutations. The development of transgenic animal models with gene mutations contributes to the study of ALS disease mechanisms and the formulation of treatment strategies, as these models recapitulate the key tissue pathology and biochemical features of ALS. Trish T. Hoang reported that *ANG* can activate the Nrf2 pathway in mouse neurons and astrocyte cell lines. This pathway provides critical cellular defence against oxidative stress. This activation primarily occurs in astrocytes but not in neurons, promoting the survival of proximal neurons with oxidative damage [98]. Ang activates the Nrf2 pathway to counteract the harmful consequences of ROS. This connection between *ANG* and Nrf2 provides a clear molecular basis for the neuroprotective effect of *ANG* and emphasizes the potential utility of Ang as a treatment for ALS.

### Clinical Heterogeneity

ALS is heterogeneous, and its clinical heterogeneity is observed in symptoms, disease progression speed, affected areas, and degree of impact. Patients may exhibit different symptoms, and the disease progression speed may vary. The affected areas may also differ; for example, in some patients, the limb muscles are affected, while in others, the respiratory muscles may be affected. The diverse mutations in the *ANG* locus also affect the heterogeneity of ALS to some extent. Identifying specific clinical features associated with *ANG* mutations is challenging.

To date, there are more than 30 mutation sites in the *ANG* gene, and different mutation sites lead to clinical heterogeneity in patients' symptoms. According to Greenway [14], bulbar ALS patients are currently more common. These patients mostly present with bulbar onset, severe symptoms, and rapid disease progression, and most die from respiratory failure. However, studies have shown that the majority of ALS patients with *ANG* mutations are still typical ALS patients [19]. Although ALS has several common clinical features, these features cannot be used as exact markers for identifying specific mutations.

Despite some progress in recent years on elucidating the association between *ANG* gene mutations and ALS, a deeper understanding of the clear correlation between mutation types and specific clinical features is still needed. Mutations at different sites may lead to different clinical manifestations and disease courses [99]. Therefore, relying on clinical features to identify specific *ANG* mutations is challenging. In addition, clinical diagnosis depends on comprehensive clinical assessment and medical history collection. However, this assessment process may require additional time and resources. It typically takes months or even years from the appearance of the initial symptoms to the diagnosis of ALS. In specific clinical scenarios, obtaining or collecting detailed medical history information in real time is difficult, which limits the ability to identify specific features related to *ANG* mutations accurately.

### Therapeutic Development

Researchers have been working tirelessly to find a cure for ALS, but currently, only two noncurative drugs are recommended for use: riluzole and edaravone [100]. Although these drugs can, to some extent, slow the progression of the disease, they cannot cure ALS. The emergence of gene therapy and the latest developments have opened up new prospects for treating ALS. Targeted gene therapy for ALS includes antisense oligonucleotides, RNA interference, CRISPR, and adeno-associated virus (AAV) [101]. Clinical trials for gene therapy targeting *SOD1*, *C9orf72*, *FUS*, and *ATXN2* are currently underway, especially since the most extensive research has focused on clinical trials of gene therapy for patients with *SOD1* mutations and *C9orf72* hexanucleotide repeat expansions [101, 102]. Additional gene therapies are under development or in preclinical trials. Although research linking *ANG* to ALS has been reported since 2006, the unclear pathogenic mechanism has prevented the development of gene therapy targeting *ANG* mutations, and clinical trials or animal experiments involving targeted drugs are needed.

Sebastià *et al*. used gene therapy in combination with Ang to establish a brain ischaemia model. They found that this treatment could enhance the reperfusion of motor neurons, thereby inhibiting neuronal degeneration and apoptosis [38]. In PD research, Steidinger *et al*. reported that Ang can promote angiogenesis and effectively inhibit the apoptosis of dopamine neurons, thus slowing neuronal degeneration [103]. Currently, relevant animal and clinical studies on the use of Ang in treating ALS are ongoing. Ronald T. Raines synthesized a variant of Ang by binding phenylboronic acid. Due to the presence of phenylboronic acid, this variant can selectively consume H2O2 at lesion sites. Upon removal of phenylboronic acid, the Ang variant reverts to Ang. In this way, the Ang variant both consumes H2O2 and replenishes Ang, suggesting its potential for use in treating ALS [104]. Recombinant *ANG* has been shown to promote the survival of motor neurons both in vitro and in vivo; therefore, the Ang protein is likely to be an effective treatment for ALS.

## 3. Perspectives: Exploration of the pathogenic mechanism of *ANG* provides new ideas for the diagnosis and treatment of ALS

### Precision Medicine

Precision medicine is an innovative approach in which personalized treatment plans are tailored according to a patient's genetic characteristics, molecular biology, and other individualized factors. Individuals are categorized into subgroups that differ in susceptibility, biology, prognosis, or response to specific treatments for a particular disease [105]. This personalized, mechanism-based therapeutic approach is primarily applied in cancer treatment and is gradually gaining greater influence. With the advancement of genetic testing technology, precision medicine is now also being applied to diagnosing and treating ALS [106]. In precision medicine for ALS, genetic sequencing technology can help identify genetic variations, providing them with more personalized treatment plans.

Over the past 30 years, three diagnostic criteria for ALS have been developed: the El Escorial criteria (1994), the revised El Escorial criteria (2000), and the Awaji criteria (2008). As the understanding of ALS has deepened, the IFCN, WFN, ALS Association, and MND Association initiated a meeting in September 2019 on the Gold Coast of Australia, proposing simpler "Gold Coast criteria" [107]. These criteria mainly focus on clinical symptoms, clinical examinations, and lower-sensitivity exclusion diagnoses. Atypical clinical presentations and neurophysiological or neuroimaging results can lead to misdiagnosis, preventing early diagnosis. With the widespread application of genomic sequencing technology, new ALS pathogenic genes have been discovered, indicating that genetic factors play a much greater role in the onset of ALS than previously thought and may also provide important means for early diagnosis of ALS. *ANG* mutations may serve as important markers for personalized treatment strategies in ALS patients. Tailoring therapies according to the specific genetic profile of patients could lead to more effective treatments.

### Identifying Modifier Genes

The pathogenic genes of ALS are constantly being explored, and currently, nearly 40 genes have been confirmed to be associated with the onset of ALS. As mentioned earlier, ALS may be a polygenic inherited disease. Pathogenic genes can be classified as major effect genes or modifier genes based on the size of the gene's phenotypic effect. The major effect genes determine the onset of the disease, while modifier genes play a role in modifying the age of onset, site of onset, progression speed, and prognosis.

The genes associated with ALS are involved in the physiological function and signalling pathways of nerve cells, and some of these genes have synergistic effects. For example, *VAPB*, *DCTN1*, and *FIG4* are involved in vesicle transport within cells; *TARDBP*, *FUS*, *ANG*, and others are involved in DNA/RNA metabolism. *TARDBP* and *FUS* have been confirmed to be associated with the onset of ALS and are involved primarily in processes such as RNA transcription, splicing, and transport [108, 109]. Most mutations in the *TARDBP* and *FUS* genes affect protein nuclear import and localization, leading to the accumulation of proteins within cells, the formation of pathological inclusions, and ultimately the eventual death of neurons [108, 110]. Expression of the *ANG* gene increases under hypoxic conditions, promoting angiogenesis, regulating ribosomal RNA transcription, and inhibiting the entry of apoptosis-inducing factors into the cell nucleus to prevent cell apoptosis [14]. Furthermore, as mentioned earlier, some patients have concurrent mutations in the *ANG* and *FUS* genes, while others have concurrent mutations in the *SOD1* and *ANG* genes. These genes may act as major effect genes and modifier genes, collectively influencing the onset and progression of ALS.

Currently, genetic testing for ALS can identify many pathogenic mutated genes. Single-gene testing technology is now mature and widely used. *C9ORF72*, *SOD1*, *TARDBP*, and *FUS* gene mutation testing are often performed on ALS patients in clinical practice. However, testing for rare genes such as the *ANG* gene is less common despite its significant role in the pathogenesis of ALS. Therefore, if conditions permit, it is best to conduct a complete ALS gene mutation test and combine it with the patient's clinical presentation for analysis. Further research may uncover modifier genes that interact with *ANG* mutations to influence disease progression. Understanding these interactions could reveal new therapeutic targets.

### RNA-based Therapies

The *ANG* gene plays an important role in RNA synthesis. When *ANG* is mutated, the production of Ang protein decreases, leading to abnormal transcription of ribosomal RNA and affecting protein nuclear import and localization, causing a reduction in neuroprotective effects and resulting in neuronal death.

RNA therapy includes a variety of drugs based on oligonucleotides, including antisense oligonucleotides (ASOs), small interfering RNAs (siRNAs), and short hairpin RNAs (shRNAs), which can be designed to selectively interact with drug targets that small-molecule drugs or monoclonal antibodies cannot currently target [111]. In addition, RNA-based therapy has the potential to modulate entire disease pathways, representing a new paradigm with unprecedented potential to serve as a disease-modifying agent for a variety of human diseases, including central nervous system (CNS) disorders [112]. Therapeutic ASOs and siRNAs are the most advanced RNA drug platforms currently in clinical development. Compared to conventional ALS therapies, they offer several advantages, such as the potential to modulate entire disease pathways and rapidly screen lead candidate drugs.

Currently, numerous clinical trials are underway worldwide to explore gene therapy for ALS, with a primary focus on targeting the SOD1, C9orf72, and ATXN2 genes. Miller *et al*. [113]. were the first to report on clinical trials involving ASOs (ClinicalTrials.gov: NCT01041222), which administered SOD1-targeting ASOs (ISIS 333611) via intrathecal injection to study their safety, tolerability, and pharmacokinetics. However, research on RNA therapy targeting *ANG* is still under development, has made limited progress, and has not yet entered clinical trials. Insights into the role of *ANG* in RNA processing may lead to the development of RNA-based therapies to restore normal RNA function in ALS patients with *ANG* mutations.

### Genetic Counselling

With improved genetic testing and understanding of *ANG* mutations, genetic counselling can play a crucial role in helping at-risk individuals make informed decisions about family planning and medical care. Genetic testing for specific pathogenic mutations in ALS helps diagnose family members with known genetic mutations (especially adolescents or those with early-onset disease) and assess risk. The risk of ALS recurrence in the family (i.e., whether another family member will develop ALS) can be assessed by determining the inheritance pattern. ALS genetic testing can provide risk information for family members who are not affected but are at risk. For families without known pathogenic mutations, genetic testing is more likely to reveal new pathogenic gene variations in ALS patients that are not detected in other at-risk family members. Once a pathogenic mutation is found, regardless of whether other family members have shown related symptoms, it is necessary to test whether they also have the same mutation [114]. Genetic testing can also be performed for SALS patients. Currently, nearly 30% of SALS patients are believed to also have genetic mutations related to FALS, including mutations in genes such as *TARDBP*, *C9ORF72*, *SOD1*, *ANG*, *FUS*, *OPTN*, and *SETX* or in genes that may be susceptibility factors for ALS (including *ATXN2*) [115]. Therefore, all ALS patients should receive genetic counselling regardless of whether they have a positive family history of ALS.

Survival of ALS patients is short. Actively exploring the pathogenic genes of ALS, clarifying the pathogenic mechanisms of rare genes (such as the *ANG* gene), conducting genetic testing for patients and other family members, and providing genetic counselling can effectively prevent the transmission of pathogenic genes. Moreover, actively following up with family members harbouring pathogenic genes but not yet symptomatic and proactively treating them to delay the onset of the disease as much as possible are highly important for improving quality of life and extending the lifespan of patients.

### Biomarker Discovery

Blood and cerebrospinal fluid (CSF) specimens are more readily available in clinical practice than histopathology specimens are, and exploring *ANG* expression in the blood and CSF of ALS patients is essential. Two of the three studies conducted on serum samples to date found elevated levels of Ang in the serum of ALS patients compared to controls [116], while another study reported the opposite finding, revealing a loss of correlation between plasma and CSF concentrations in ALS patients, indicating tissue-specific dysregulation [72]. Although no significant case-control differences were reported in a small study of Ang levels in the CSF of ALS patients [16], Moreau *et al*. revealed an ALS-specific lack of Ang upregulation during hypoxemia by examining Ang CSF levels associated with respiratory status [117]. Given the conflicting results reported to date, the utility of this protein as a valuable biomarker for ALS warrants further investigation. A research team [118] evaluated differences in Ang levels in CSF samples from 88 Italian patients with ALS and/or FTD and 46 controls. In addition, they analysed the relationship between clinical parameters and Ang levels. They found no significant difference in Ang levels between the case and control groups. According to multivariate regression models, CSF Ang concentrations were significantly greater in patients with FTD or ALS-FTD than in ALS patients without dementia and controls (p< 0.001). In ALS-FTD patients, no correlation was found between Ang levels and clinical parameters, including age, presence of C9orf72 repeats, or body mass index (BMI) [118]. These findings highlight the role of Ang as a CSF biomarker for identifying patients with ALS who also have FTD and suggest that Ang should be further explored as a potential biomarker for ALS.

*ANG*-related research may lead to the discovery of biomarkers that can aid in early ALS diagnosis and monitoring disease progression. Biomarkers could facilitate clinical trials and patient care. Furthermore, longitudinal studies are needed to determine whether combining protein-based candidate biomarkers with neurophysiological and neuroimaging-derived biomarkers would enhance diagnostic sensitivity and accuracy, aid in monitoring disease progression, or predict the prognosis of ALS.

### Multidisciplinary Approaches

Multidisciplinary care is a comprehensive medical model that involves collaborative efforts across multiple disciplines, such as genetics, neurology, and molecular biology, and is crucial for the overall understanding of and the search for new treatment approaches for ALS associated with *ANG*. Genetics research provides an in-depth understanding of the disease mechanism, neurology research focuses on the clinical manifestations and treatment of the disease, and molecular biology research aids in identifying potential therapeutic targets and developing new treatment strategies. By collaborating with these diverse disciplines, a more comprehensive understanding of *ANG*-related ALS can be achieved, leading to more effective treatment options for patients. Genetics plays a crucial role in understanding ALS associated with *ANG*. Through family studies and genome sequencing, specific genetic variations associated with the risk of ALS can be identified, providing important clinical information for neurologists. Furthermore, genetic research can reveal the impact of different genetic variations on treatment response, providing a basis for personalized treatment.

In a multidisciplinary team, neurologists' clinical experience and expertise can provide important clinical data to guide molecular biology research and the development of new treatment methods. Additionally, molecular biology research provides a vital foundation for identifying new treatment approaches. By analysing the function and regulatory mechanisms of the *ANG* gene, researchers can uncover disease pathways and potential therapeutic targets associated with this gene. Using the findings of molecular biology research, researchers can develop novel treatment strategies targeting the *ANG* gene, such as gene editing techniques, RNA interference, and protein-based drugs.

### Patient Advocacy and Support

As genetic discoveries continue, patient advocacy and support organizations can play vital roles in raising awareness, providing resources, and fostering a sense of community among ALS patients and their families.

ALS is a rare but fatal neurological disease, and patients and their families often face immense physical and psychological pressure. Despite the increasing number of genetic discoveries, ALS support organizations play a crucial role in helping patients and families cope with this challenge. ALS support organizations can increase public awareness of ALS through various channels and collaborate with medical professionals to provide training and education for better diagnosis and treatment of ALS patients. Additionally, ALS support organizations can provide various resources to help patients and families better cope with ALS. These resources may include information manuals, online support groups, mental health services, family care guides, etc. Furthermore, they may also provide financial aid and assistance for patients to access medical equipment and services. By providing these resources, ALS support organizations can help patients and families better understand and manage ALS, reduce their burden, and improve their quality of life. ALS support organizations strive to create a united, supportive, and understanding community environment, allowing patients and families to share experiences, exchange emotions, and find mutual support.

### International Collaboration

Collaboration between global researchers, clinical practitioners, and institutions is crucial to accelerating the understanding and development of effective treatment methods for ALS related to *ANG*. By fostering interdisciplinary and international cooperation, the scientific and medical community can unravel the complexities of *ANG*-associated neurodegeneration and expedite the translation of research findings into innovative therapies. Ultimately, this collaborative approach has the potential to significantly impact the lives of ALS patients and their families worldwide.

Collaboration between researchers from different countries and institutions is essential for understanding the genetic, molecular, and cellular mechanisms underlying ALS related to *ANG*. By sharing data, resources, and expertise, scientists can collectively investigate the complex interactions between *ANG* and disease progression. This collaboration can involve joint research projects, data-sharing initiatives, and regular scientific meetings to exchange findings and ideas. For example, geneticists, molecular biologists and neuroscientists can collaborate to identify specific mutations in *ANG* that contribute to ALS and elucidate the pathways through which *ANG* dysfunction leads to neurodegeneration. Furthermore, clinical practitioners are vital in advancing research on *ANG*-related ALS by providing access to patient populations and contributing valuable clinical insights. Collaborative efforts between clinicians and researchers can facilitate the collection of genetic and clinical data from ALS patients with *ANG* mutations, enabling the identification of common disease patterns and the evaluation of potential therapeutic interventions. Multicentre clinical trials and patient registries can be established through international collaborations to gather large-scale data and validate the efficacy of targeted treatments.

In addition to collaborative research, developing effective treatment methods for *ANG*-related ALS requires interdisciplinary cooperation. Bioinformaticians, computational biologists, and biostatisticians can work together to analyse large-scale genomic and clinical datasets and identify genetic modifiers and biomarkers associated with *ANG* mutations and disease progression. By integrating computational modelling and experimental validation, interdisciplinary teams can accelerate the translation of genetic insights into therapeutic strategies. Moreover, international collaboration is essential for the preclinical and clinical development of potential treatments for *ANG*-related ALS. Academic and industry partnerships can facilitate the discovery and optimization of therapeutic agents targeting *ANG*, such as RNA-based therapies or small-molecule inhibitors. Collaborative efforts can expedite the preclinical testing of candidate drugs in cellular and animal models and the design of clinical trials to assess their safety and efficacy in ALS patients.

## 4. Discussion

ALS is a rapidly progressing neurodegenerative disease with an unclear aetiology and pathogenesis. The treatment drugs currently in use cannot achieve a cure and can only alleviate symptoms. As research has progressed, an increasing number of ALS disease-causing genes have been discovered. Ang is an angiogenic molecule that can act on rRNA transcription in endothelial cells and plays a role in angiogenesis [89]. In recent years, many studies have reported the presence of *ANG* loss-of-function mutations in ALS patients [15]. Moreover, researchers have shown that Ang is strongly expressed in the spinal cord [75] and that Ang may play an essential role in coordinating the function of motor neurons by acting on endothelial cells and motor neurons. Previous studies have explored the role of Ang in angiogenesis and maintaining vascular stability; combined with its effects on motor neurons, these findings suggest the importance of Ang in motor neuron development. Previously, we described the *ANG* mutations detected in ALS patients. According to statistics, ALS patients with *ANG* mutations account for approximately 0.86% of all ALS patients, 2.0% of FALS patients, and 0.7% of SALS patients [13, 24]. *ANG* mutations are relatively common in FALS patients, and we further explored the proportion of *ANG* mutations in each ALS subtype. Current studies have not analysed *ANG* mutations in patients with different types of ALS. However, according to the study by Greenway [14], bulbar ALS patients are currently more common than nonbulbar ALS patients, but according to other studies, most ALS patients with *ANG* mutations are still typical ALS patients [19]. Combining these reports, we analysed the possibility that this difference occurred because patients with classic ALS are the most common, and the proportion of patients with classic ALS caused by *ANG* mutations is more significant. However, we further analysed the data of ALS patients with *ANG* mutations reported to date. Compared with ALS patients without *ANG* mutations, patients with mutations were more critically ill, and most of them were diagnosed with medulla oblongata ALS and died of respiratory failure within 2 years of onset. We found that *ANG* mutations may increase the severity and heterogeneity of ALS. However, the mechanism of *ANG*-induced ALS is still being explored. Theories of polygenic inheritance, loss of the neuroprotective function of Ang, oxidative stress, and inhibition of rRNA synthesis are widely recognized. Despite the pathogenicity of most *ANG* variants, this has not been demonstrated.

Several studies have linked *ANG* to the pathology of upper and lower motor neurons (MNs) in diseases such as ALS and neuronal development and neuroprotection [119]. *ANG* is expressed in response to hypoxia [75], and previous reports have shown that *ANG* has neuroprotective functions. *ANG* has a neuroprotective effect on MNs under hypoxic conditions, but the ANG-ALS variant lacks this function. The combination of *ANG* and *VEGF* has a mild synergistic effect on MN survival, suggesting that *VEGF* and *ANG* may act through different pathways in terms of neuroprotection [20]. Furthermore, although evidence of oxidative damage is widespread in the pathogenesis of ALS, the ultimate trigger for elevated ROS levels remains unknown, leading to speculation about whether oxidative stress is a primary cause or a secondary consequence of the disease. ALS is a multifactorial disease, and oxidative stress may be the underlying biological basis for multiple seemingly unrelated stressors. Oxidative stress not only affects the systemic health of ALS patients but also impacts the integrity of motor neurons. Lipid peroxidation products such as HNE or IsoPs are highly neurotoxic and may be produced centrally and peripherally due to oxidative stress [120]. However, they can affect vulnerable MNs in the CNS through damage to the brain microvascular endothelial cells of the blood-brain barrier, leading to their breakdown [121]. If oxidative stress is determined to be a key factor in the pathogenesis of *ANG*-mutant ALS, innovative clinical trials will be needed to study oxidative stress successfully and develop more effective treatment and prevention strategies. With a deeper understanding of the mechanisms by which *ANG* gene mutations lead to cellular oxidative stress, the treatment and management of ALS will be based on biology. These factors may lead to proactive and appropriate treatment methods to slow or even halt the progression of ALS.

As a member of the pancreatic RNase family, Ang is essential for biological activity, and its association with RNA processing and neurodegenerative diseases has been established [122]. Further studies have demonstrated that Ang promotes angiogenesis for treating neurological diseases and stimulates cell survival and growth mechanisms in neurons and axons. The mechanism through which angiogenin functions in endothelial cells and MNs may be related to its ability to mediate ribosome biogenesis. When Ang is translocated to the cell nucleus, it is concentrated in the nucleolus [60], the site of rRNA transcription and ribosome assembly. Ribosome synthesis and assembly are among the most complex and energy-consuming tasks that cells perform. Each functional ribosome consists of 79 ribosomal proteins and 4 rRNAs, which account for approximately 80% of the total cellular RNA, and mRNA-encoding ribosomal proteins may constitute >20% of the total cellular mRNA pool [40]. Ang is transported to the nucleolus of nerve cells, where it binds to the ribosomal DNA (rDNA) promoter region and stimulates rRNA transcription to synthesize many ribosomes to meet the needs of nerve cell growth and transport in the axon [86]. The role of Ang in motor neurons is also related to rRNA transcription. When the *ANG* gene is mutated, the mutant form of Ang can cause defects in transcription pathways that may lead to insufficient ribosome synthesis, thereby affecting the vitality of motor neurons. Previous studies have suggested that ALS is caused by a hypoxic response that leads to MN degeneration, and cells adapt to hypoxic environments by activating hypoxia-inducible factor-1 (HIF-1), which in turn regulates the expression of hypoxia response genes [15]. Mutations in the *ANG* gene coding region in ALS patients lead to insufficient or defective Ang, resulting in insufficient rRNA transcription in MNs. Although *ANG* expression is not altered in transgenic ALS mouse models or sporadic ALS patients, culturing MN cells under hypoxic conditions can increase HIF-1 and *ANG* expression; the addition of recombinant *ANG* can protect motor neurons from hypoxia-induced damage, whereas *ANG* silencing leads to an increase in hypoxia-induced cell death [17]. Further studies have shown that *ANG* is expressed in growth cones and axons, the spinal cord, dorsal root ganglion neurons, and neurons derived from P19 cells undergoing mitosis, suggesting that *ANG* plays an important role in axon growth [19, 75]. Dysfunction in Ang is not only caused by heterozygous missense mutations in the *ANG* gene coding region, polymorphisms in the *ANG* promoter region, or reduced expression due to other genetic and environmental factors but also leads to abnormal RNA splicing, ribosome assembly, and gene transcription, creating an unhealthy environment for MNs. The absence of Ang may accelerate or cause MN degeneration, thereby promoting the onset and development of ALS. We speculate that Ang protein therapy may be beneficial for treating ALS.

A positive *ANG* genetic test can accelerate the diagnosis of ALS, allowing patients to start receiving drug treatment early in the disease course. In 1985, Ang was first isolated from the culture medium of colon adenocarcinoma cells by Vallee et *al*. at Harvard Medical School [123]. Ang is a protein with strong angiogenic activity. Currently, Ang drugs are primarily used in the field of tumour treatment. *ANG* plays dual roles in promoting tumour cell proliferation and angiogenesis in tumour growth and is also an essential permissive factor for VEGF, aFGF, bFGF, and EGF in promoting angiogenesis. Therefore, inhibiting *ANG* in tumour treatment has advantages over previous single angiogenic factor inhibitors. By promoting rRNA transcription through nuclear translocation, *ANG* promotes tumour cell proliferation and angiogenesis [124]. The nuclear translocation inhibitor of *ANG*, called neomycin, has been studied internationally as an anticancer drug due to its low toxicity, high specificity, and high efficiency and has entered the development stage [124-126]. Research has shown that Ang can increase the blood supply to ischaemic areas by promoting tissue angiogenesis, establishing new collateral circulation, improving ischaemic symptoms, and inhibiting neuronal degeneration and apoptosis, thus treating neurological diseases.

The best method to elucidate the role of Ang in ALS is to create and characterize *ANG* KO mice. Humans have only one *ANG* gene, but mice have six [127]. It is not feasible to knock out all of these genes simultaneously, as they are distributed over >80 kb. Mouse *ANG*1 is the major form and orthologue of the human gene [128]. Therefore, knocking out mouse *ANG*1 may be useful for studying the function of human angiogenin. Because the loss of mouse angiogenin-1 function may be embryonically lethal, a conditional KO model can be created to study the role of angiogenin-1 in MNs at different stages of development. If the absence of *ANG*1 leads to MN degeneration, *ANG*1 JO mice could be used for ALS drug screening. In the future, additional animal experiments and clinical trials are needed to explore the therapeutic effect of Ang on ALS to identify new treatment options. In the future, additional gene therapies based on *ANG* mutations are expected to be explored, combining advances in the pathogenic mechanism of *ANG*, CNS targeting, gene delivery, and gene editing and knockdown technologies to open up new perspectives for ALS treatment.

In the previous section, we noted that there is still a lack of specific drugs available for treating ALS; therefore, caring for ALS patients is very important, as it can improve their quality of life and slow the progression of the disease. Previous case reports have shown that ALS patients with *ANG* mutations mostly have bulbar-onset ALS, and most patients have respiratory dysfunction, ultimately dying from respiratory paralysis or pulmonary infections. Early identification of *ANG* gene mutations and exploration of its pathogenic mechanism in ALS patients are expected to assist in the care of ALS patients. Doctors and family members should regularly monitor the respiratory condition of ALS patients with *ANG* mutations and provide respiratory assistance devices at night to improve their breathing function. In addition, attention should also be given to providing effective nutritional support for ALS patients with *ANG* mutations, which can improve their nutritional status and enhance their quality of life.

This paper has several limitations. First, due to limited space, part of the discussion was not included, but this does not affect academic reading comprehension. In addition, most of the studies included in this search were limited to English-language publications and were mainly cross-sectional studies; therefore, selection bias can easily occur during the literature search, and some relevant literature may have been omitted. However, we included as many studies as possible to explore the role of *ANG* mutations in ALS pathogenesis. In addition, because ALS itself is a rare disease, the number of ALS patients with *ANG* mutations is small compared with the number of patients with SOD1 mutations, leading to a lack of studies. Moreover, most related studies are limited to population genomic studies, with few mechanistic studies. In addition, there are no animal models of *ANG* mutation or *ANG*-targeted therapy. Therefore, this paper focuses more on the possible pathogenic mechanism of *ANG* mutations into ALS, and there is insufficient space to discuss potential therapeutic strategies involving *ANG* genes. However, relevant systematic reviews are needed to better evaluate the role of *ANG* mutations in the pathogenesis of ALS and to explore potential targeted intervention strategies.

## 5. Conclusion

There are many possible mechanisms for the occurrence and development of ALS induced by *ANG*. The more widely recognized mechanisms are the polygenic inheritance theory, loss of the neuroprotective function of Ang, oxidative stress, and inhibition of rRNA synthesis. CSF and blood, especially CSF, are ideal clinical specimens that can reflect pathological changes in the CNS related to neurodegenerative diseases. Detecting *ANG* expression is of particular significance for diagnosing ALS in the future, especially for ALS patients with the medulla oblongata type and the possibility of severe respiratory complications, and it is important to use genes for early diagnosis and treatment. However, according to recent studies, *ANG* mutations lack specificity. For example, Ang can also be expressed in PD patients and patients with cognitive impairment. In addition, several healthy individuals carrying *ANG* mutations have been reported. In the future, additional clinical and animal studies are needed to further explore the pathogenesis of *ANG* mutations to improve the early diagnosis and treatment of this disease.

## Data Availability

The original contributions presented in the study are included in the article, and further inquiries can be directed to the corresponding authors.
